# Protecting isolated reptile populations outside their main area of distribution: a predictive model of the Dice snake, *Natrixtessellata*, distribution in the Czech Republic

**DOI:** 10.3897/BDJ.11.e114790

**Published:** 2023-12-28

**Authors:** Jan Chmelař, Petr Civiš, David Fischer, Daniel Frynta, Lenka Jeřábková, Veronika Rudolfová, Ivan Rehák

**Affiliations:** 1 Department of Zoology, Faculty of Science, Charles University, Viničná 7, Prague, Czech Republic Department of Zoology, Faculty of Science, Charles University, Viničná 7 Prague Czech Republic; 2 Department of Ecology, Faculty of Environmental Sciences, Czech University of Life Sciences Prague, Kamýcká 129, Prague, Czech Republic Department of Ecology, Faculty of Environmental Sciences, Czech University of Life Sciences Prague, Kamýcká 129 Prague Czech Republic; 3 Mining Museum Příbram, Hynka Kličky 293, Příbram, Czech Republic Mining Museum Příbram, Hynka Kličky 293 Příbram Czech Republic; 4 Nature Conservation Agency of the Czech Republic, Kaplanova 1931/1, Prague, Czech Republic Nature Conservation Agency of the Czech Republic, Kaplanova 1931/1 Prague Czech Republic; 5 Prague Zoo, Prague, Czech Republic Prague Zoo Prague Czech Republic

**Keywords:** conservation, monitoring, species management, river phenomenon

## Abstract

Marginal populations of animals are highly susceptible to environmental pressures associated with climatic changes. Understanding their distribution and ecological requirements is, thus, essential for the development of efficient conservation strategies. The dice snake, *Natrixtessellata*, is listed as critically endangered in the Czech Republic. In certain regions (Bohemia and Silesia), its populations are located beyond the northern border of the continuous range of the species, while the south Moravian populations are connected to it. Based on the statewide database of the Czech Nature Conservation Agency, we created a predictive model and determined key factors influencing the species distribution. The most relevant factors were: watercourses and bodies, average annual temperatures, altitude, slope inclination and precipitation seasonality. The model fits the presence records well and is applicable in both theory and practice of the species conservation – for example, focusing faunistic research to certain areas, critical analysis of controversial presence reports and as an input for species management in the form of repatriation and introduction.

## Introduction

In ecology, predictive models are becoming increasingly popular as a tool for complex distribution analysis and identification of key climatic and geographical factors (Elith et al. 2006, Civiš 2013). As the number of studies increases, the focus is not only on the distribution of plants, but is expanding to animals, including reptiles (Kaliontzopoulou et al. 2008, Hosseinian Yousefkhani et al. 2013, Sillero and Carretero 2013, Oraie et al. 2014, Wirga and Majtyka 2015, Vargas-Ramírez et al. 2016, Petrosyan et al. 2020, Chmelař et al. 2020, Srinivasulu et al. 2021), predictions of invasive species spreading (Pyron et al. 2008, Jarnevich et al. 2018) and even in relation to climate change (Dubey et al. 2012). Especially small isolated populations are presumed to be most threatened by habitat erosion due to the climate change (Sinervo et al. 2010).

In the Czech Republic, the dice snake, *Natrixtessellata* (Laurenti, 1768) is generally rare and declining, as a result of habitat degradation (Pecina 1991, Velenský et al. 2011) and introduction of the invasive American mink (*Neovisonvison*) that predates on *N.tessellata* (Kapler 1994, Mikátová et al. 2001, Šváb 2003, Musilová and Zavadil 2011). The habitat fragmentation due to roads and cycle paths that often lead close to the river banks and either degrade the habitat directly or block the migration route between feeding and wintering and reproduction sites are also factors. (Telenchev et al. 2017). According to legislative regulations in the Czech Republic, *N.tessellata* is listed amongst the critically endangered species according to the Ministry of the Environment of the Czech Republic and the current Red List of amphibians and reptiles for the Czech Republic lists the species as endangered (Jeřábková et al. 2017).

The recent distribution of *N.tessellata* in the Czech Republic is the result of post-glacial expansion of the species from south-glacial refuges, while the isolated Bohemian and German populations are presumed to be from Holocene climatic optimum (Guicking and Joger 2011); expansion of *N.tessellata* from glacial refuges was also documented for Asian populations (Jablonski et al. 2023). The origin and distribution in the Czech Republic are similar to the distribution of the European green lizard (*Lacertaviridis*) and the distribution of these species significantly overlaps mostly in river valleys in Bohemia, but also in southern parts of Moravia (Moravec 2015, Chmelař et al. 2020). In Bohemia, Silesia and Germany, *N.tessellata* distribution is isolated from the continuous range of the species and these individual populations are mostly isolated from each other (Gruschwitz and Günther 1996, Mikátová et al. 2001, Vlček et al. 2010, Guicking and Joger 2011, Moravec 2015). Molecular data confirmed genetic affinities of Bohemian populations (samples from rivers Berounka and Ohře) to those in neighbouring parts of *N.tessellata* distribution in Germany, Bulgaria, Romanian Donau Delta, Slovakia and Serbia (Guicking et al. 2009). On the other hand, samples from the population near Havířov show a similar haplotype to populations in Hungary, southern Austria and Slovenia (Jablonski et al. 2014). Furthemore, recent molecular data confirm interspecific hybridisation within the genus *Natrix* (Asztalos et al. 2021, Schöneberg et al. 2023).

Since 2007, the Czech Nature Conservation Agency has been monitoring the presence of *N.tessellata* in order to obtain the most up-to-date and comprehensive picture of the species distribution. While certain places of occurrence have traditionally been well known in the long term since 1790 (Lindaker 1790, Štěpánek 1949, Moravec 2015), no published data are available in other areas of the Czech Republic, where natural conditions do not exclude the presence of *N.tessellata*.

The focus of this recent paper is to review yet unpublished Nature Conservation Agency faunistic reports, to analyse available distribution records, to identify the key factors affecting the distribution of *N.tessellata* and to create a predictive model of the species distribution in the Czech Republic. We intend to help to prioritise the monitoring effort (to focus on places where the predicted probability of presence is high, but no real presence has been recorded). At the same time – by comparing the predictive model with the known distribution of *N.tessellata* in the Czech Republic (based on critically evaluated published and our own data) – to evaluate the usefulness of creating predictive distribution models for the theory and practice of conservation and species management in particular.

## Material and methods

As a source of *N.tessellata* presence sites, we used a statewide database maintained by the Nature Conservation Agency of the Czech Republic. The database contained 660 records from the whole territory of the Czech Republic collected within the period from 1895 to 2014, with only 102 records being older than 1980.

For modelling purposes, a total of 73 layers were created for the Czech Republic: the lowest, highest and average temperatures for individual months (36 layers in total), precipitation in individual months (12), bioclimatic variables according to worldclim.org methodology (Table 1) (19), altitude, surface exposure, human footprint, slope, road network, watercourses and -bodies (including a 200 m buffer on each side of the watercourse or -body) (Civiš 2013).

For modelling via the MaxEnt interface, WorldClim Worldwide database is routinely used as the source of climate variables. However, this database uses data from only two meteorological stations for the whole Czech Republic (Hijmans et al. 2005), which is why it was unsuitable for our research and we have created layers for the bioclimatic variables manually.

All layers were created in ArcGis 9.3 (ESRI ArcGIS 2008) in the 2D coordinate system S-JTSK Krovak East-North. The layers of climate variables were created, based on the Climate Atlas of Czechia (Czech Hydrometeorological Institute 2007), which includes data from 1961 to 2000.

Variables containing maximum, minumum and average temperatures and precipitation in individual months were not included in the model because of their high intercorrelation. In similar cases, a careful interpretation is recommended for making possible implications for species conservation (Syfert et al. 2013). These data were also already included in the WorldClim bioclimatic variables.

The climatic variables were screened for intercorrelation in ENM Tools (Warren et al. 2010), resulting in correlation matrices for the Pearson correlation coefficient "r", Pearson coefficient of determination "r^2^" and Variance Inflation Factor "VIF" (Suppl. material 1). Variables with r > 0.8, r > 0.8 and VIF > 10 were considered heavily intercorrelated (Pradhan 2016) and removed from the model not to be used alongside variables with which they were closely correlated. The final model included the following variables: altitude, aspect, human footprint, road network, BIO 1, BIO 2, BIO 3, BIO 4, BIO 6, BIO 7, BIO 8, BIO 9, BIO 12, BIO 15, slope, watercourses and -bodies.

The Predictive Distribution Model was created using MaxEnt software (Phillips et al. 2023), the output being a GIS document in .asc format. This software was specifically chosen to work well with presence-only data (Elith et al. 2006, Hernandez et al. 2006). As the *N.tessellata* presence prediction value, the “Logistic threshold” was defined, i.e. the optimisation between the sensitivity of the model and the location of all real places of presence in the predicted areas. MaxEnt model was run in three replications and automatically cross-validated. All other settings in the model were set to default.

## Results

**Figure 1.**
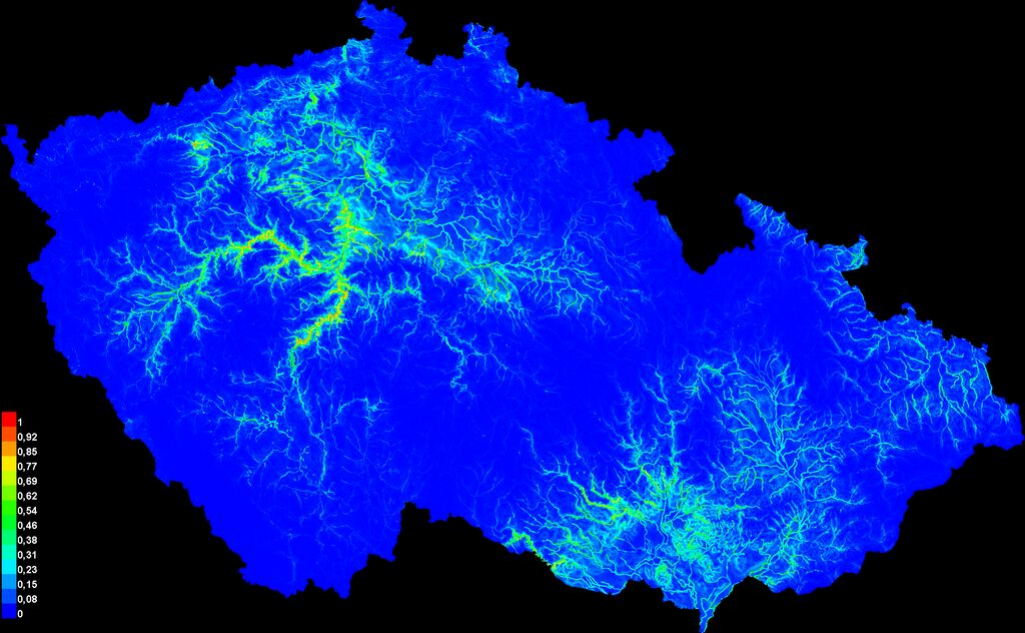
Predicted distribution of the dice snake, *Natrixtessellata* (Laurenti, 1768), in the Czech Republic.

**Figure 2.**
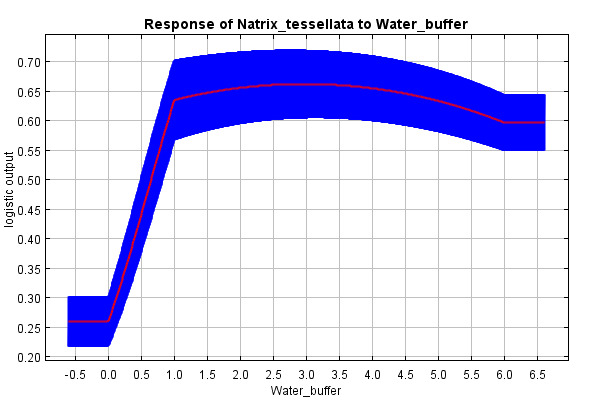
Response curve of the watercourses and -bodies variable (including 200 m buffer around courses, y-axis: 1-3 watercourses ascending with course size, 4-6 waterbodies, ascending with body size).

**Figure 3.**
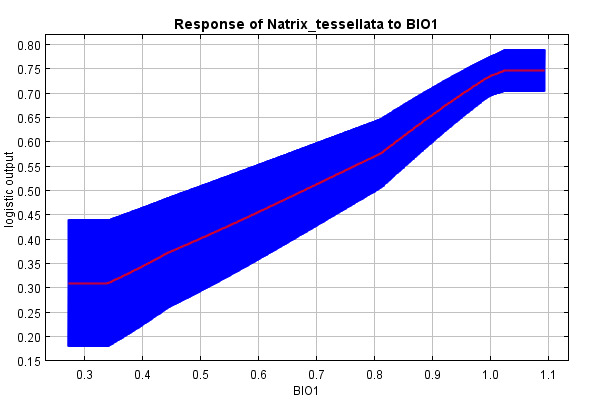
Response curve of the annual average temperature (°C / 10).

**Figure 4.**
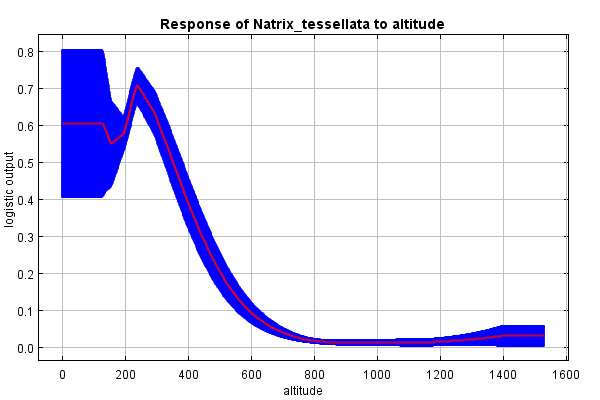
Response curve of altitude (m a.s.l.).

**Figure 5.**
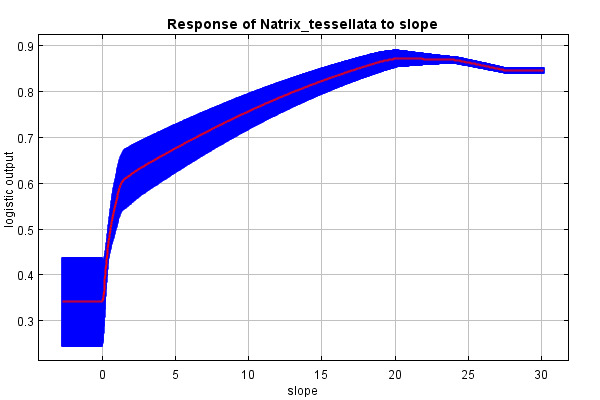
Response curve of slope (°).

**Figure 6.**
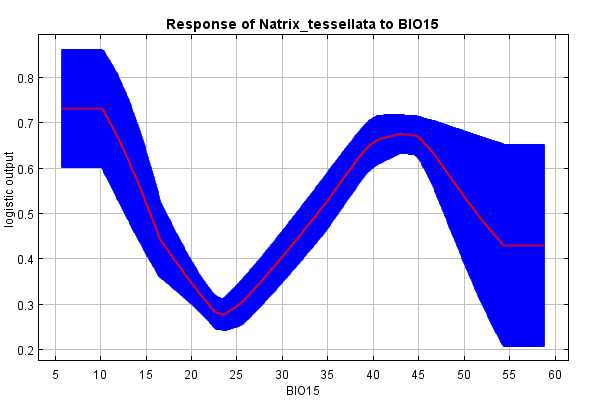
Response curve of BIO 15 - Precipitation Seasonality (Seasonality Index).

The predictive strength of the model (mean AUC) was 0.92 (92% of actual presence records were above the prediction threshold) (Suppl. material 2). The mean “logistic threshold” prediction, when the sensitivity of the model is equal to its specificity, was 0.151. Therefore, any higher value means that the model predicts *N.tessellata* presence (see Fig. 1). The most important contributors to the resulting model were: Watercourses with 200 m buffer - Water_buffer (explaining 36.7% variability), BIO 1 – Average annual temperature (18.7%), altitude (11.6%), slope (11.4%) and BIO 15 - Precipitation seasonality (9.6%) Figs 2, 3, 4, 5, 6.

According to the model, the probability of presence of *N.tessellata* is highest in areas up to 200 m from watercourses and -bodies (Fig. 2) in areas with annual temperatures of 10-11°C (Fig. 3). The probability of presence sharply drops in altitudes 250-400 m a.s.l. (Fig. 4) and is highest in areas with slope inclination of 20-25° (Fig. 5). The probability of presence is also highest in areas with the Precipitation Seasonaility index (Walsh and Lawler 1981) of 0-10 and 40-45 (Fig. 6).

## Discussion

The predictive strength of the model was very high (mean AUC = 0.92). Only 8% of the actual real presence points were located below the predicted occurrence threshold, including several remote presence points from areas where *N.tessellata* occurrence is unlikely – for example, area near Česká Třebová and Zábřeh in the East Bohemia/West Moravia or Volary and Vimperk in the South-western Bohemia. These points are listed in the national database as taken over from sources labelled as controversial. The number of these points is negligible and we consider it a price for a large data sample from a wide spectrum of informants. Since the database is only open for authorised zoologists, included records are reliable and the size of the input dataset contributes to the prediction strength (Hernandez et al. 2006, Merow et al. 2013).

The probability of presence by annual average temperature is highest at 10-11°C. This result is in full accordance with the published data and with the fact that significant areas with higher temperatures are hotspots of the actual *N.tessellata* distribution (Mikátová et al. 2001, Moravec 2015). So far, there is no indication from the core area of distribution that higher temperatures are restraining the presence of the species. The average and maximum temperatures on the majority of the area are gradually increasing from 1961 (Zahradníček et al. 2020). The increase seems to be even faster in years 2011 to 2019 and these increases are most severe in the already warmest areas of the Czech Republic with the increase as high as 0.43°C per 10 years. This could mean significant future changes of suitable habitats for many species, including reptiles.

The predicted presence probability increased with proximity to watercourses and -bodies. This corresponds with publications about populations at or beyond the northern edge of the species continuous area of distribution (Mikátová et al. 2001, Moravec 2015) and, apart from species ecology, this can be explained by the fact that the Czech *N.tessellata* populations are linked to the so-called river phenomenon (Jeník and Slavíková 1964, Vannote et al. 1980, Ložek 1988, Ward 1998, Mikátová et al. 2001). The specific geology and temperature, water and air regime of deeply incised river valleys allow the occurrence of thermophilic organisms beyond the northern boundary of their continuous range on river slopes with southern exposition.

Increasing probability of prediction with decreasing altitude and increasing slope inclination supports the suspected link of the distribution to river valleys and its importance was closely followed by altitude. Near the northern edge of the species distribution range, *N.tessellata* inhabits mostly altitudes of 200-350 m a.s.l. (Mikátová et al. 2001, Moravec 2015). This corresponds with the model, where the probability of prediction sharply drops above approximately 400 m a.s.l., although *N.tessellata* can be found even higher in the mountains in the southern parts of its range (Piemonte, Italy up to 2000 m a.s.l., Austria up to 840 m a.s.l., Bulgaria up to 1420 m a.s.l., Asia up to 2800-3000 m a.s.l. (Rehák 1992, Nistri et al. 1997, Grillitsch and Cabela 2001, Stojanov et al. 2011).

Precipitation was expected to influence the species distribution, given that Czech *N.tessellata* populations are piscivorous and closely related to rivers and waterbodies as a source of prey. The highest probability of presence in areas with a Seasonality Index (Walsh and Lawler 1981) of 0-10 (precipitation spread throughout the year) and 40-45 (rather seasonal with a short drier season) corresponds to the avoidance of excessively humid locations for reproduction and wintering (Mikátová et al. 2001, Moravec 2015). There is also a strong assumption of intercorrelation of average annual precipitation with average annual temperatures (Schultz and Halpert 1993).

The link to the river phenomenon was well documented for *N.tessellata* distribution in Bohemia (Ložek 1988, Mikátová et al. 2001, Moravec 2015). The regional pattern of distribution suggests historical contraction of the range following the period of the Holocene climatic optimum with *N.tessellata* populations surviving in these refugia (Guicking and Joger 2011).

**Figure 7.**
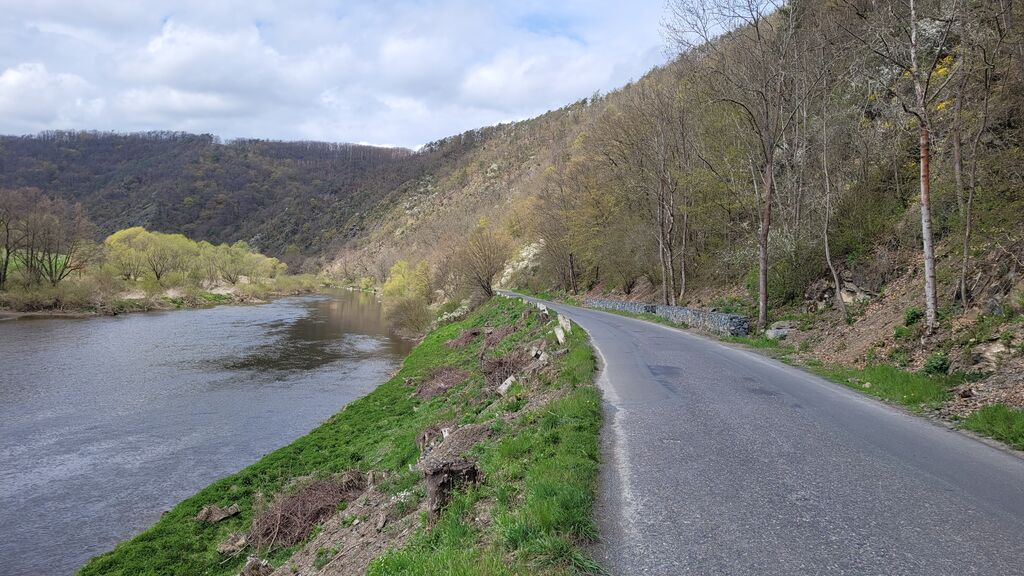
Picture of a *N.tessellata* biotope in Bohemia (Nezabudické skály Natural Reserve).

**Figure 8.**
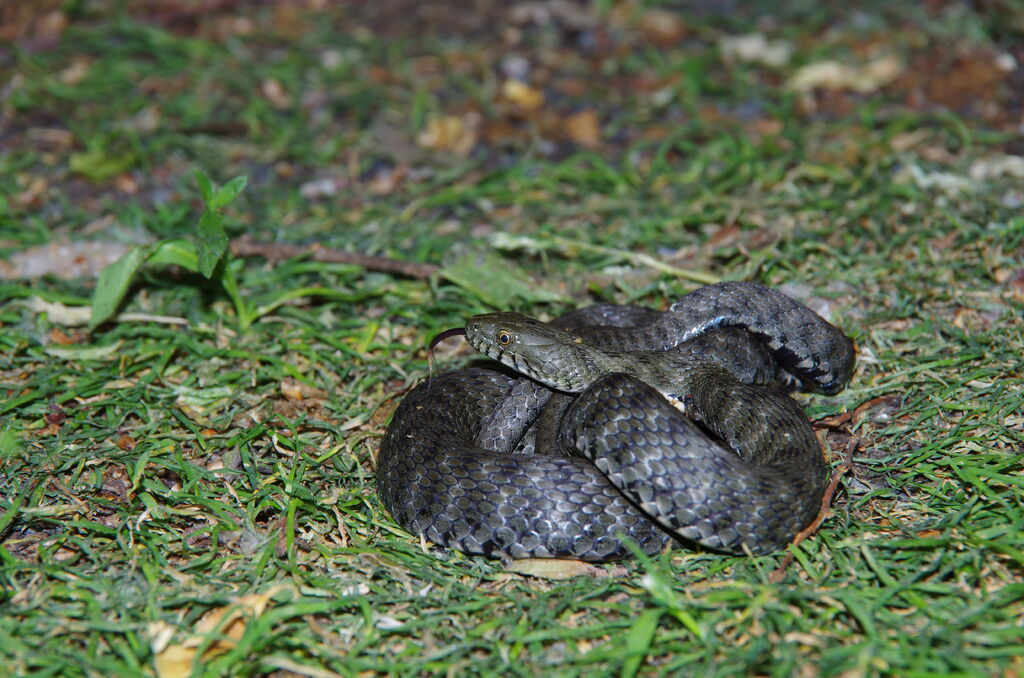
*Natrixtessellata* individual from a population in Bohemia (Nezabudické skály Natural Reserve).

**Figure 9.**
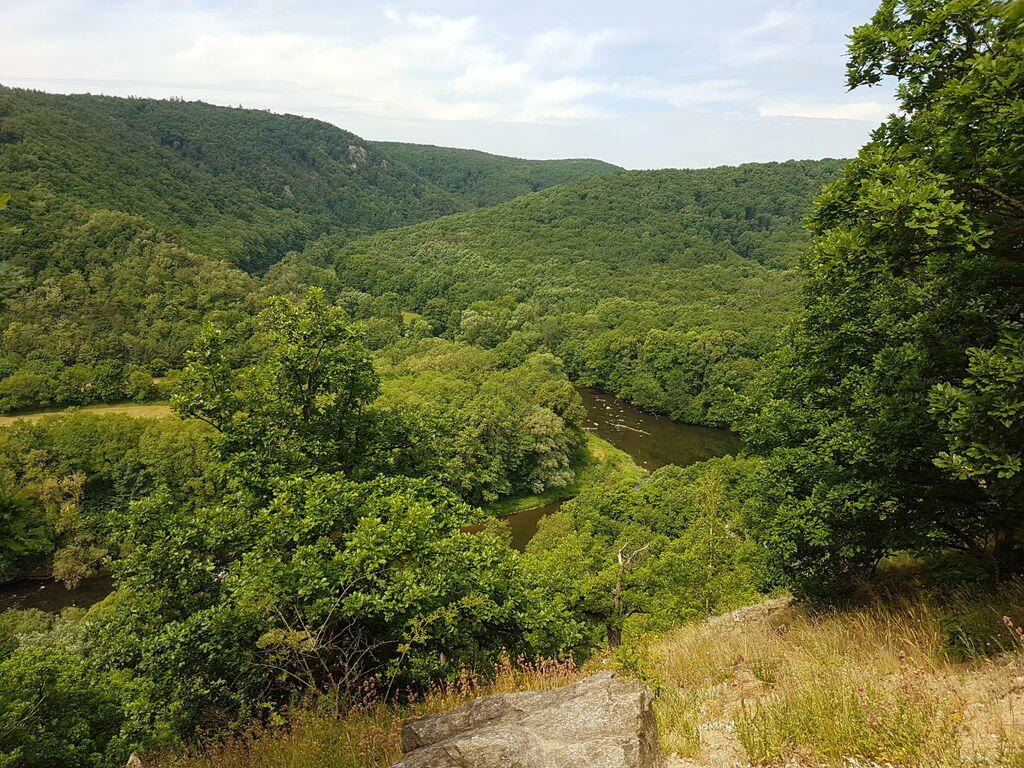
Picture of *N.tessellata* biotope in South Moravia (Podyjí National Park).

**Figure 10.**
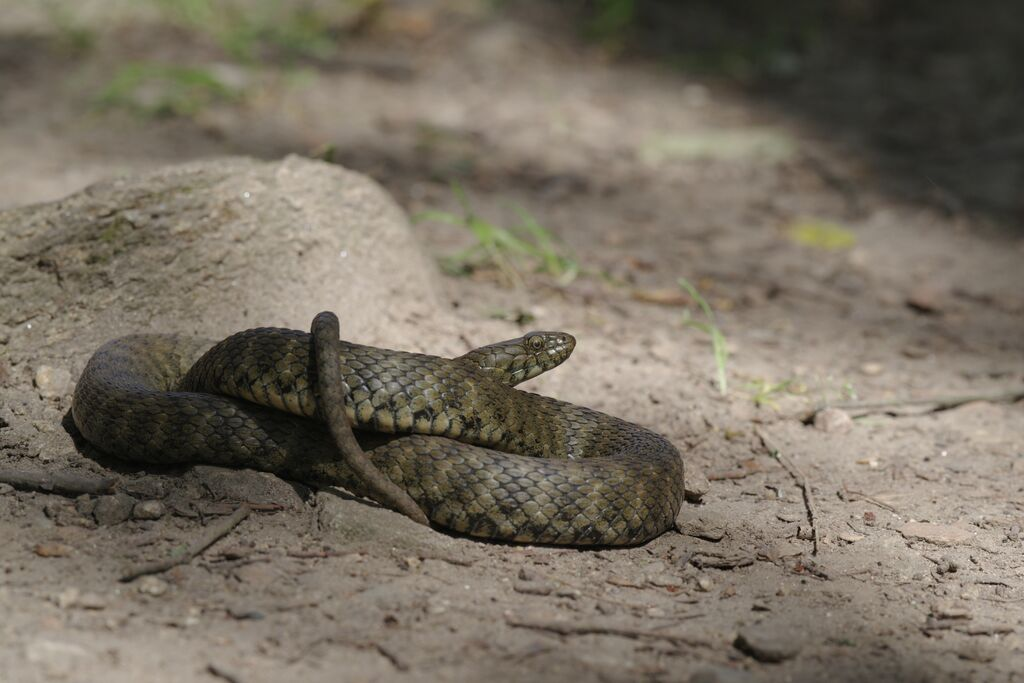
*Natrixtessellata* individual from a population in South Moravia (Podyjí National Park).

The resulting map of the species presence in Bohemia is in full compliance with the occurrence of the river phenomenon in the Czech Republic. In this model, the precipitation variable explains a large portion of variability, but only when combined with other significant variables. Examples of *N.tessellata* from localities in Bohemia (isolated from the continuous area of species distribution) and from localities in South Moravia (north-western border of the continuous species range) are shown in Figs 7, 8, 9, 10.

The most recently published map of distribution (Moravec 2015, Šandera 2023) is in good consistency with our predictive model, but since it is a square network map without precise locations and coordinates, a more detailed comparison is problematic – especially with regard to the extraordinarily variable geomorphological relief of the Czech Republic and to the distribution of *N.tessellata* in the Czech Republic, where there is a strong correlation with the geomorphological relief variability – in this case, the network mapping easily includes the areas where the species is not present.

The link of Bohemian *N.tessellata* populations to specific microclimatic parameters and terrain morphology suggests that the most effective conservation strategy should be protecting their actual and predicted habitats. Additionally, the habitats linked to river phenomenon seem to be amongst the most resistant to climate change which otherwise poses a major threat to reptile populations (Sinervo et al. 2010, Dubey et al. 2012). Since climatic data from a large area have lower resolution, we recommend to analyse climatic parameters and their changes on a smaller scale, for example, several populations within areas with the highest temperature increases.

## Conclusions

We consider the result of finding large areas with high probability of modelled prediction where real occurrence is not reported to be extraordinarily important. Here, we see the need to direct species monitoring to these places and search for historical data. These sites should be considered as a matter of priority for possible repatriation efforts and for conservation management by species introduction if the nature conservation authority decides for them. Additionally, these areas might be possible corridors for migration. In recent history, reptile species have been observed to occur beyond the northern range of their continuous areas (Jablonski et al. 2014, Rehák et al. 2022) with the possibility of both natural migration and human introduction. This expansion could also provide opportunity for hybridisation with related species (Asztalos et al. 2021, Schöneberg et al. 2023). The areas pinpointed from the model should also be subject to analysis of human footprint and possible dangers, for example, new cyclist corridors are being constructed along major rivers leading to heightened mortality and turning promising migration corridors into ecological traps.

The comparison of our predictive model and real distribution shows that the predicted and real distribution are almost fully accordant. Thus, a creation of predictive distribution models is a helpful instrument to facilitate monitoring and conservation efforts.

## Supplementary Material

4409CCE7-35C8-5BAD-91B2-1ED319E0FD93 10.3897/BDJ.11.e114790.suppl1 Supplementary material 1 Bioclimatic variable corellation matrix Data type statistic Brief description This file contains correlation matrices for the Pearson correlation coefficient (r), Pearson coefficient of determination (r^2^) and Variance Inflation Factor (VIF) for bioclimatic variables used in the model. File: oo_946645.xlsx https://binary.pensoft.net/file/946645 Jan Chmelař, Veronika Rudolfová

6FD530D6-4D1D-5E35-98E8-CFB5CD3B0E85 10.3897/BDJ.11.e114790.suppl2 Supplementary material 2 MaxEnt model output Data type distribution Brief description Pdf. file showing cross-validated output of triplicate MaxEnt model. File: oo_946646.pdf https://binary.pensoft.net/file/946646 Authors team

## Figures and Tables

**Table 1.** Bioclimatic variables according to worldclim.org methodology.

Variable	Description
BIO1	Annual Mean Temperature
BIO2	Mean Diurnal Range (Mean of monthly (max temp - min temp))
BIO3	Isothermality (BIO2/BIO7) (* 100)
BIO4	Temperature Seasonality (standard deviation *100)
BIO5	Max Temperature of Warmest Month
BIO6	Min Temperature of Coldest Month
BIO7	Temperature Annual Range (BIO5-BIO6)
BIO8	Mean Temperature of Wettest Quarter
BIO9	Mean Temperature of Driest Quarter
BIO10	Mean Temperature of Warmest Quarter
BIO11	Mean Temperature of Coldest Quarter
BIO12	Annual Precipitation
BIO13	Precipitation of Wettest Month
BIO14	Precipitation of Driest Month
BIO15	Precipitation Seasonality (Coefficient of Variation)
BIO16	Precipitation of Wettest Quarter
BIO17	Precipitation of Driest Quarter
BIO18	Precipitation of Warmest Quarter
BIO19	Precipitation of Coldest Quarter
